# High anaemia and iron deficiency prevalence among pregnant women living in low groundwater iron areas of Bangladesh

**DOI:** 10.1186/s12889-024-20480-2

**Published:** 2024-11-06

**Authors:** Mohammed Imrul Hasan, Shamim Ahmed, Alistair R. D. McLean, A. M Quaiyum Rahman, Mohammad Saiful Alam Bhuiyan, S. M. Mulk Uddin Tipu, Sabine Braat, Shams El Arifeen, Jena D. Hamadani, Sant-Rayn Pasricha, Eliza M. Davidson

**Affiliations:** 1 Maternal and Child Health Division, International Centre for Diarrhoeal Disease Research, GPO Box 128, Dhaka, 1000 Bangladesh; 2 Population Health and Immunity Division, The Walter and Eliza Hall Institute of Medical Research, Melbourne, Australia; 3 Medical Biology, Faculty of Medicine Dentistry and Health Sciences, The University of Melbourne, Melbourne, Australia; 4 Methods and Implementation Support for Clinical and Health (MISCH) Research Hub, Faculty of Medicine, Dentistry and Health Sciences, The University of Melbourne, Melbourne, Australia; 5 Diagnostic Haematology, The Royal Melbourne Hospital, Parkville, Australia

**Keywords:** Anaemia, Iron deficiency, Pregnancy, Drinking water iron, Bangladesh

## Abstract

**Background:**

Anaemia is a significant public health concern in Bangladesh, yet data on the prevalence of anaemia in pregnancy and the contribution of iron deficiency are limited. Given the reliance on groundwater for drinking, a better understanding of the role of drinking water iron in anaemia aetiology is also required to inform anaemia prevention strategies.

**Methods:**

This cross-sectional study enrolled 1500 pregnant women from Narayanganj district, Bangladesh, during their second or third trimester. Anaemia and iron status were described and their relationship with drinking water iron assessed using regression analyses.

**Results:**

The prevalence of anaemia was 38% (95% confidence interval, CI: 35%, 40%), with 10% (95% CI: 9%, 12%) experiencing moderate-to-severe anaemia. Iron deficiency affected 48% (95% CI: 45%, 50%) of participants. Although drinking water iron concentrations were generally low (median: 0 mg/L; interquartile range: 0–1), high concentrations (≥ 2 mg/L) were associated with increased ferritin levels but did not significantly impact iron deficiency (95% CI: 0·73, 1·02) or anaemia (95% CI: 0·79, 1·17) prevalence. Iron deficient women had a 1·86 (95% CI: 1·61, 2·15) adjusted prevalence ratio for anaemia and a 4·22 (95% CI: 2·89, 6·17) adjusted prevalence ratio for moderate-to-severe anaemia, compared to iron replete women.

**Conclusions:**

Anaemia and iron deficiency are highly prevalent among pregnant women in Narayanganj. These findings challenge the assumption of low iron deficiency prevalence throughout Bangladesh and highlight iron deficiency in pregnancy as a potentially underrecognized public health problem, particularly in regions with low drinking water iron. Further research is needed to clarify the contribution of drinking water iron to iron deficiency and anaemia in Bangladesh.

**Trial registration:**

The study is registered with the Australian and New Zealand Clinical Trials Registry (ACTRN12621000982819, registered 26/07/2021)

**Supplementary Information:**

The online version contains supplementary material available at 10.1186/s12889-024-20480-2.

## Background

Anaemia in pregnancy is a widespread condition, with 37% of all pregnant women estimated to be anaemic [1]. This burden is disproportionately experienced in low- and middle-income countries. The primary cause of anaemia is iron deficiency [2]. Pregnant women are particularly susceptible to iron deficiency due to the high iron requirements of pregnancy [3]. Anaemia in pregnancy contributes substantially to maternal morbidity and impaired quality of life [4]. Maternal anaemia has also been linked to adverse birth outcomes, including low birthweight and preterm delivery [5–8].

In Bangladesh, anaemia is highly prevalent in women of reproductive age, with 42% found to be anaemic in the 2011 Demographic and Health Survey (DHS) [9]. Within a small subset of pregnant women, 50% were anaemic. However, the prevalence of iron deficiency was low, with 7% of non-pregnant, non-lactating women reported to be iron deficient in the 2011 national micronutrient survey [10]. Importantly, this survey did not assess iron deficiency in pregnant women. Overall, there is a paucity of data on the prevalence of anaemia, and the contribution of iron deficiency to anaemia, in pregnant Bangladeshi women.

The relationship between iron status and anaemia in Bangladesh is complicated by the presence of iron in the groundwater. A hydrochemical survey conducted in 1998–1999, found numerous areas of elevated groundwater iron (≥ 2 mg/L) throughout Bangladesh, but particularly in the north [11]. Groundwater is the most common drinking water source in Bangladesh, with 73% of urban households and 96% of rural households relying on hand operated wells (tubewells) for drinking water [12]. High groundwater iron is thought to contribute to dietary iron intake and consequently, it has been proposed that iron deficiency is not a substantial public health problem in Bangladesh [10]. In support of this, a national cross-sectional study demonstrated a geographical overlap between the distribution of low groundwater iron in the hydrochemical survey, [11] and iron deficiency prevalence [13]. Similarly, no iron deficiency was found in women of reproductive age living in a high groundwater iron area of Northern Bangladesh [14]. While these epidemiological studies suggest that the groundwater iron in Bangladesh is bioavailable, few studies have assessed the direct relationship between iron in drinking water on iron and/or anaemia status. A better understanding of the role of drinking water iron in the aetiology of anaemia in Bangladesh is required to inform setting-specific anaemia prevention strategies.

To address these knowledge gaps, we determined the prevalence of anaemia and iron deficiency in 1500 pregnant women from the Narayanganj district of Bangladesh, and assessed the relationships between drinking water iron, iron status and anaemia.

## Methods

### Study design and participants

This cross-sectional study took place between October 2021 and April 2022, during which a total of 1500 pregnant women in their second or third trimester were recruited. The study was conducted across three adjacent upazilas (sub-districts) – Rupganj, Sonargaon and Bandar, within the Narayanganj district of Bangladesh. These upazilas are located approximately 30–35 km outside of Dhaka. This region is currently undergoing a transition from a rural, agrarian economy to a semi-urban, industrialized one. This study area was selected as we recently implemented an iron intervention trial in children living in Rupganj upazila, [15, 16] and therefore have a strong presence in the community and an established relationships with local health authorities.

In the government health system of Bangladesh, Family Welfare Assistants (FWAs) visit assigned households every one-two months to identify and register pregnancies. Our study Field Research Assistants (FRAs) routinely collected pregnancy records from the FWAs’ registers and contacted potentially eligible women to arrange home visits (see participant flow diagram in Supplementary Fig. 1). At home visits, FRAs confirmed that women met the inclusion criteria, which required being 13–32 weeks pregnant (calculated from first day of last menstrual period, LMP) and residing within the study catchment area. After obtaining informed consent, participants were asked to provide information on their socio-demographics, household assets, income, nutritional knowledge, and smoking status. Iron levels were measured in their main drinking water source using the HACH iron test kit, Model IR-18B.

The following day, participants attended a nearby Union Family Welfare Centre, where a study physician performed a physical examination and collected information on obstetric and antenatal care practice history, iron-folic acid (IFA) use, and delivery preparedness. A water, sanitation and hygiene (WASH) and Edinburgh postnatal Depression Scale (EPDS) survey were completed. A 5ml venous blood sample was collected from each participant, and haemoglobin level was measured using a HemoCue® Hb 301. The anaemia status of participants was explained, and all participants were provided IFA tablets and instructions for use, as per standard-of-care.

### Laboratory procedures

Following collection of the venous blood, samples were transferred to the local field laboratory, where serum was separated and stored for ferritin and C-reactive protein (CRP) analysis. Samples were initially stored at -20 °C but periodically transferred to -80 °C storage at the central lab in Dhaka. Ferritin was measured by electrochemiluminescence immunoassay (ECLIA) using the Roche Diagnostics automated immunoassay Cobas e601 analyser. CRP was measured by immunoturbidimetric method using the Roche Diagnostics Cobas c311 analyser.

Ethics approval was obtained from the Ethical Review Committee of International Centre for Diarrhoeal Disease Research, Bangladesh (PR-20125) and the Human Research Ethics Committee of the Walter and Eliza Hall Institute, Melbourne, Australia (21/5). The study is registered with the Australian and New Zealand Clinical Trials Registry (ACTRN12621000982819, registered 26/07/2021). The research was conducted in strict accordance with the Declaration of Helsinki.

### Classification of anaemia and iron status

Anaemia status was classified as follows: no anaemia (haemoglobin ≥ 110 g/L), mild anaemia (haemoglobin < 110 and ≥ 100 g/L), moderate anaemia (haemoglobin < 100 and ≥ 70 g/L), and severe anaemia (haemoglobin < 70 g/L). Iron status was classified as iron replete (ferritin ≥ 15 µg/L, or ferritin ≥ 30 µg/L in the presence of inflammation, C-reactive protein, CRP > 5 mg/L) or iron deficient (ferritin < 15 µg/L, or ferritin < 30 µg/L in the presence of inflammation, CRP > 5 mg/L).

### Statistical analysis

Regarding sample size, we planned to screen 1500 pregnant women, evenly distributed across the Upazilas, with 40% selected from the second and third trimesters, and the remaining 20% chosen competitively. With a minimum sample size of 600 women per trimester across all Upazilas, the precision of the two-sided 95% confidence interval (CI) of the true underlying prevalence is at most ± 4% (assuming prevalence of 50%) using the Wald method. Anticipated precisions include ± 3·8% for anaemia prevalence (assuming prevalence of 35%, 95% CI: 31·2%, 38·8%), ± 2·4% for moderate/severe anaemia (10%, 95% CI: 7·6%, 12·4%), and ± 3·9% for iron deficiency (40%, 95% CI: 36·1%, 43·9%).

Analyses included all recruited pregnant women. Cohort characteristics, including drinking water iron, were summarised. The number and proportion of women with anaemia and iron deficiency was derived, alongside two-sided 95% confidence intervals using the Wald method. Univariable and multivariable linear and logistic regression analyses were performed to determine associations between drinking water iron exposures (water iron concentration, low < 2 mg/L/ high ≥ 2 mg/L), iron status outcomes (ferritin concentration, iron deficient/replete), and anaemia outcomes (haemoglobin level, anaemic/non-anaemic). In addition to unadjusted analyses, analyses were adjusted for potential confounders. The adjustment set was selected a priori based on a causal diagram (Supplementary Fig. 2): age (continuous), Upazila (Rupganj/ Sonargaon/ Bandar), gravidity (primigravida: first pregnancy/ multigravida: ≥ 2 previous pregnancies), gestational age in weeks (continuous, calculated from last menstrual period date), education level (primary or less/ secondary/ tertiary), mid-upper arm circumference (MUAC, continuous), income quintile ((quintile 1 (relative poorest)/ quintile 2/ quintile 3 (relative middle)/ quintile 4/ quintile 5 (relative wealthiest)), smokeless or chewing tobacco use (no/ yes), indoor smoke exposure (cooking indoors with solid fuels, e.g., coal, charcoal, wood, straw, crops, animal dung; no/ yes) and history of IFA use (no/ yes). To explore effect modification of the relationship between drinking water iron and iron status and anaemia outcomes by self-reported drinking water source (tubewell/piped: piped into dwelling, piped into yard, public tap), we included drinking water source and an interaction term between drinking water source and drinking water iron (low < 2 mg/L/ high ≥ 2 mg/L) as covariates in the model.

Univariable and multivariable linear and logistic regression analyses were also performed to determine associations between iron status exposures (ferritin concentration, iron deficient/replete) and anaemia outcomes (haemoglobin level, anaemic/non-anaemic). Multivariable models included the adjustment set listed above, along with drinking water iron (low < 2 mg/L/ high ≥ 2 mg/L) (Supplementary Fig. 3). Ferritin and drinking water iron were log base 2 transformed before fitting the models. For logistic regression models, prevalence ratios were obtained using marginal effects and corresponding confidence intervals using the delta method (STATA “adjrr” command) [17].

Data were analyzed using STATA Version 17.0 (StataCorp, Statistical Software: College Station, TX) and DAGitty Version 3.0 was used to draw a directed acyclic graph to guide the adjustment set in our multivariable analyses [18].

## Results

### Cohort characteristics

In this cohort of 1500 pregnant women, 500 were recruited from Rupganj, Bandar and Sonargaon Upazilas each (Table 1). The median age of women was 23 years (interquartile range, IQR: 19–26), and 26% (384/1500) were ≤ 19 years. The median gestation age was 24 weeks (IQR: 19–28); 60% (897/1500) were in their second trimester (13–25 weeks) and 40% (603/1500) were in their third trimester. Two thirds of women were multigravida (1000/1500, 67%) and the median interpregnancy interval for these women was 56 months (IQR: 33–82). Half had a secondary school education (744/1497, 50%) and most were housewives (1458/1497, 97%). Median monthly family income was 17,000 Bangladeshi Taka (IQR: 12,000–30,000, 165 US$ [conversion October 2021]). Smokeless or chewing tobacco was used by only 2% (25/1497), while no women reported smoking cigarettes or cigars. Indoor smoke exposure through cooking with solid fuels (e.g., coal, charcoal, wood, straw, crops, animal dung) occurred for 33% (499/1500).

**Table 1:** Cohort characteristics of pregnant women in the Narayanganj district, Bangladesh

		**Total (*N* = 1500)**
Enrolment Upazila	Rupganj	500 (33·3%)
	Sonargaon	500 (33·3%)
	Bandar	500 (33·3%)
Age (years)		23 (19–26)
	≤ 19 years	384 (25·6%)
	20–34 years	1071 (71·4%)
	≥ 35 years	45 (3·0%)
Gravidity	Primigravida	500 (33·3%)
	Multigravida	1000 (66·7%)
Parity^a^	Nullipara	80 (8·0%)
	Primipara	601 (60·1%)
	Multipara	319 (31·9%)
Pregnancy interval (months)^b^		56 (33–82)
	Short interval (< 18 months)	71 (9·0%)
	Long interval (≥ 18 months)	715 (91·0%)
Gestational age (weeks)^c^		24 (19–28)
	Trimester 2 (13–25 weeks)	897 (59·8%)
	Trimester 3 (26–32 weeks)	603 (40·2%)
MUAC (cm)		26·2 [3·4]
	MUAC > 23 cm	1204 (80·3%)
	MUAC ≤ 23 cm	296 (19·7%)
BMI (kg/m^2^)		24·6 [4·1]
Level of education^d^	No education	48 (3·2%)
	Primary (1–8 years)	705 (47·1%)
	Secondary (9–12 years)	677 (45·2%)
	Tertiary (> 12 years)	67 (4·5%)
Employment status^e^	Housewife	1458 (97·4%)
	Manual job	11 (0·7%)
	Non-manual job	25 (1·7%)
	Other	3 (0·2%)
Smokeless or chewing tobacco	No	1472 (98·3%)
	Yes	25 (1·7%)
Indoor smoke exposure^f^	No	998 (66·7%)
	Yes	499 (33·3%)
Taking oral iron supplements	No	431 (28·7%)
	Yes	1069 (71·3%)
Antenatal care received^g^	No	521 (34·7%)
	Yes	979 (65·3%)
Husband’s age (years)		30 (27–35)
Husband’s level of education^d^	No education	165 (11·0%)
	Primary (1–8 years)	728 (48·6%)
	Secondary (9–12 years)	488 (32·6%)
	Tertiary (> 12 years)	116 (7·8%)
Husband’s employment status^e^	Unemployed	41 (2·7%)
	Manual job	620 (41·4%)
	Non-manual job	732 (48·9%)
	Other	104 (6·9%)
Monthly family income (Taka)^h^		17,000 (12,000–30,000)
Number living in household^i^		4 (3–6)

Data are presented as median (lower quartile – upper quartile), mean [Standard Deviation], or n (%)

*MUAC* Mid-upper arm circumference, *BMI* Body Mass Index

^a^*N* = 1000

^b^*N* = 786. Represents the interval between the current and last pregnancy in multigravida women whose previous pregnancy outcome was a stillbirth or live birth

^c^Gestational age calculated from last menstrual period date

^d^*N* = 1497

^e^*N* = 1497. “Manual job” refers to manual labor such as farming, fishing, or rickshaw pulling etc. Non-manual job refers to roles requiring specialized skills, such as garment work, carpentry, administration, healthcare, business ownership, teaching, or engineering)

^f^*N* = 1497

^g^Indicates women who attended at least 1 antenatal healthcare visit during the current pregnancy

^h^*N* = 1496. Monthly family income is in Bangladeshi Taka (1USD = 103 Bangladeshi Taka, conversion October 2021, Xe.com)

^i^*N* = 1497. Reflects the total number of people living in the household

Regarding antenatal care, 65% (979/1500) had visited a health care provider prior to recruitment, with their first visit occurring at a median of 13 weeks’ gestation (IQR: 8–17); however, 35% (521/1500) had not yet received any antenatal care (42%, 357/897 in the second trimester; 24%, 146/603 in the third trimester). When asked about IFA supplement use during this pregnancy, most reported taking IFA (1069/1500, 71%), with 60% (898/1500) reporting that they took it “most days” (4–6 times per week).

### Prevalence of anaemia and iron deficiency

The mean haemoglobin level in this cohort was 111.8 g/L (standard deviation, SD: 10.6) and 38% (567/1500, 95% CI: 35%, 40%) were anaemic; 28% (413/1500; 95% CI: 25%, 30%) were mildly anaemic, 10% (152/1500; 95% CI: 9%, 12%) were moderately anaemic, and 0.1% (2/1500; 95% CI: 0.01%, 0.3%) were severely anaemic (Table 2). Nearly half, 48% (714/1498; 95% CI: 45%, 50%), of women were iron deficient, and 24% (361/1498; 95% CI: 22%, 26%) were both iron deficient and anaemic. Importantly, iron deficiency was highly prevalent amongst women who were moderately–severely anaemic at 79% (122/154; 95% CI: 73%, 86%). The prevalence of anaemia and iron deficiency was similar across Upazilas (Supplementary Table 1).

**Table 2:** Anaemia and iron deficiency status overall and by trimester

	**Total** **(*N* = 1500)**	**Second trimester** **(*N* = 897)**	**Third trimester** **(*N* = 603)**
Anaemia status			
No anaemia	933 (62·2%; 60·0%, 64·7%)	584 (65·1%; 62·0%, 68·2%)	349 (57·9%; 53·9%, 61·8%)
Anaemia	567 (37·8%; 35·3%, 40·3%)	313 (34·9%; 31·8%, 38·0%)	254 (42·1%; 38·2%, 46·0%)
Mild anaemia	413 (27·5%; 25·3%, 29·8%)	238 (26·5%; 23·6%, 29·4%)	175 (29·0%; 25·4%, 32·6%)
Moderate anaemia	152 (10·1%; 8·6%, 11·7%)	73 (8·1%; 6·3%, 9·9%)	79 (13·1%; 10·4%, 15·8%)
Severe anaemia	2 (0·1%; 0·01%, 0·3%)	2 (0·2%; 0·0%, 0·5%)	0·0 (0·0%)
Iron deficiency status^a^			
Iron replete	784 (52·3%; 49·8%, 54·9%)	537 (59·9%; 56·7%, 63·1%)	247 (41·1%; 37·2%, 45.0%)
Iron deficient	714 (47·7%; 45·1%, 50·2%)	360 (40·1%; 36·9%, 43·3%)	354 (58·9%; 55·0%, 62·8%)
Iron deficiency anaemia status^a^	361 (24·1%; 21·9%, 26·3%)	181 (20·2%; 17·6%, 22·8%)	180 (30·0%; 26·2%, 33·6%)

Data are presented as n (%; 95% confidence interval)

Anaemia status is classified as follows: no anaemia (haemoglobin ≥ 110 g/L), mild anaemia (haemoglobin < 110 and ≥ 100 g/L), moderate anaemia (haemoglobin < 100 and ≥ 70 g/L), and severe anaemia (haemoglobin < 70 g/L)

Iron status is classified as iron replete (ferritin ≥ 15 µg/L, or ferritin ≥ 30 µg/L in the presence of inflammation, C-reactive protein, CRP > 5 mg/L) or iron deficient (ferritin < 15 µg/L, or ferritin < 30 µg/L in the presence of inflammation, CRP > 5 mg/L)

Iron deficiency anaemia is defined as haemoglobin < 110 g/L combined with ferritin < 15 µg/L, or ferritin < 30 µg/L in the presence of inflammation (CRP > 5 mg/L)

^a^*N* = 1498 total, with *N* = 897 in the second trimester and *N* = 601 in the third trimester

Anaemia and iron deficiency prevalence were higher in the third trimester of pregnancy (Supplementary Fig. 4), with 42% (254/603; 95% CI: 38%, 46%) anaemic, 59% (354/601; 95% CI: 55%, 63%) iron deficient, and 30% (180/601; 95% CI: 26%, 34%) both anaemic and iron deficient in the third trimester. Among women in the third trimester who were moderately-severely anaemic (13%, 79/603; 95% CI: 11%, 16%), most were also iron deficient (86%, 68/79; 95% CI: 76%, 93%) (Supplementary Table 2).

### Associations between drinking water iron, iron status and anaemia

Iron levels measured in drinking water ranged from 0-7 mg/L (Supplementary Table 3), 56% (842/1500) of women had a drinking water iron measurement of 0 mg/L. According to the threshold of 2 mg/L, [19] 13% (197/1500) of women had high drinking water iron levels (≥ 2 mg/L); ranging from 4% (19/500) in Rupganj, 16% (79/500) in Bandar, to 20% (99/500) in Sonargaon Upazila.

**Fig. 1:**
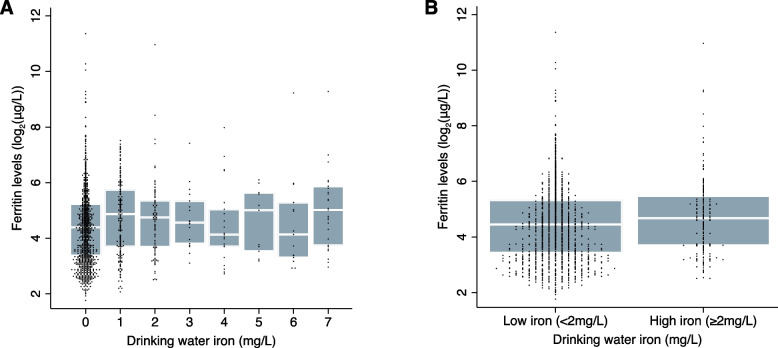
Boxplot of the relationship between drinking water iron and serum ferritin concentration (*N* = 1498). Panel 1**a** displays ferritin concentrations across all measured drinking water iron levels, while panel 1**b** compares ferritin concentrations between low and high drinking water iron levels. Ferritin concentrations are transformed to log base-2 due to their positively skewed distribution. The boxes represent the interquartile range (25th to 75th percentiles), the horizontal white line within each box represents the median, and individual dots represent the actual data points

The relationship between drinking water iron and ferritin concentration is depicted in Fig. 1. The variability of ferritin levels within drinking water iron strata was high (Spearman’s rank correlation coefficient: 0.10), ferritin levels were slightly lower in the low versus high drinking water iron group (median 21.9 μg/L, IQR 10.8–40.1; versus median 25.5 μg/L, IQR 13.0–44.5). In adjusted regression analyses, women with high drinking water iron had higher ferritin levels (adjusted mean difference 0·28; 95% CI 0·09, 0·47) relative to low drinking water iron (Table 3). High drinking water iron was associated with an adjusted prevalence ratio of 0·86 in iron deficiency, but the data were compatible with both a moderate reduction and slight increase in prevalence (95% CI: 0·73, 1·02).

**Table 3:** Associations between drinking water iron levels and ferritin, iron deficiency, haemoglobin concentration and anaemia (*N* = 1500)

	**Ferritin level (log**_**2**_**(μg/L))**^**†**^		**Iron deficiency**^**a†**^		**Haemoglobin (g/L)**		**Anaemia**^**b**^	
	**Unadjusted mean difference (95% CI); *p*-value**	**Adjusted mean difference (95% CI); *p*-value**	**Unadjusted prevalence ratio (95% CI); *p*-value**	**Adjusted prevalence ratio (95% CI); *p*-value**	**Unadjusted mean difference (95% CI); *p*-value**	**Adjusted mean difference (95% CI); *p*-value**	**Unadjusted prevalence ratio (95% CI); *p*-value**	**Adjusted prevalence ratio (95% CI); *p*-value**
Drinking water iron (log_2_(mg/L)) ^c^	0·08 (0·05, 0·12); < 0·001	0·09 (0·05, 0·12); < 0.001	0·95 (0·92, 0·98); 0·005	0·94 (0·90, 0·97); 0·001	0·30 (0·01, 0·60); 0·04	0·24 (-0·08, 0·57); 0·14	0·97 (0·93, 1·01); 0·13	0·97 (0·93, 1·02); 0·22
Drinking water iron								
Low (< 2 mg/L)	Reference	Reference	Reference	Reference	Reference	Reference	Reference	Reference
High (≥ 2 mg/L)	0·22 (0·03, 0·42); 0·03	0·28 (0·09, 0·47); 0·003	0·90 (0·76, 1·07); 0·24	0·86 (0·73, 1·02); 0·08	0·63 (-0·95, 2·22); 0·43	0·82 (-0·77, 2·40); 0·31	0·98 (0·80, 1·19); 0·82	0·96 (0·79, 1·17); 0·69

Linear regression models were used to assess the association between drinking water iron (discrete or categorical) and continuous outcomes (ferritin, haemoglobin), deriving mean differences. Ferritin was log base-2 transformed prior to analysis. For categorical outcomes (iron deficiency, anaemia), logistic regression models were used, with prevalence ratios obtained using marginal effects and delta method for confidence intervals. Adjusted models included: Upazila, age, gestation week, gravidity, mid-upper arm circumference, education, tobacco use, indoor smoke exposure, income quintile, and iron folic acid use. R-squared values for adjusted models were ferritin (0.15, 0.15), iron deficiency (0.08, 0.09), haemoglobin (0.06, 0.06), anaemia (0.03, 0.03), respectively

*CI* Confidence Interval

^**†**^*N* = 1498

^a^Iron status is classified as iron replete (ferritin ≥ 15 µg/L, or ferritin ≥ 30 µg/L in the presence of inflammation, C-reactive protein, CRP > 5 mg/L) or iron deficient (ferritin < 15 µg/L, or ferritin < 30 µg/L in the presence of inflammation, CRP > 5 mg/L)

^b^Anaemia status is classified as no anaemia (haemoglobin ≥ 110 g/L) or anaemia (haemoglobin < 110 g/L)

^c^Drinking water iron is transformed to log base-2 due to a positively skewed distribution, with observations of no detected water iron set to half the detection limit (0.125mg/L). The estimate represents the change (relative for ferritin, iron deficiency, anaemia; absolute for haemoglobin) associated with a two-fold increase in drinking water iron

**Fig. 2:**
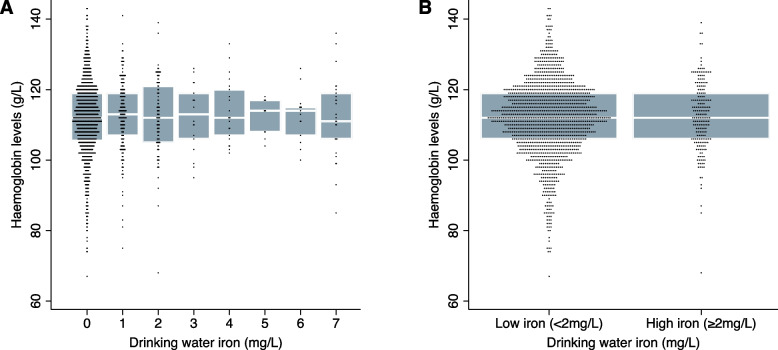
Boxplot of the relationship between drinking water iron and haemoglobin concentration (*N* = 1500). Panel 2**a** displays haemoglobin concentration across all measured drinking water iron levels, while panel 2**b** compares haemoglobin concentrations between low and high drinking water iron levels. The boxes represent the interquartile range (25th to 75th percentiles), the horizontal white line within each box represents the median, and individual dots represent the actual data points

The relationship between drinking water iron and haemoglobin concentration is shown in Fig. 2. There was no discernible difference in haemoglobin concentration with increasing drinking water iron level (Spearman’s rank correlation coefficient: 0·04), and haemoglobin levels were similar in the low (mean 111·7 g/L, SD 10·7) and high (mean 112·4 g/L, SD 9·8) drinking water iron group. In adjusted regression analysis, there was no statistically significant association between high drinking water iron and haemoglobin level (adjusted mean difference: 0·82 g/L; 95% CI: -0·77, 2·40) relative to low drinking water iron (Table 3) or anaemia prevalence (adjusted prevalence ratio: 0·96; 95% CI: 0·79, 1·17).

Tubewells/boreholes were used as the main drinking water source for 40% (605/1500) of all women, ranging from 12% (61/500) of those living in Rupganj, to 40% (198/500) in Bandar and 69% (346/500) in Sonargaon (Supplementary Table 3). Other drinking water sources included piped water into the house or yard, or public taps. High drinking water iron levels (≥ 2 mg/L) were more common in those who reported using tubewells/boreholes as their primary drinking water source (24%) compared to other sources (5–7%). To further assess the relationship between drinking water iron levels and iron/anaemia status, an interaction term was fitted to allow the effect to vary by main drinking water source. Among women who relied on tubewells/boreholes, ferritin and haemoglobin levels still did not appear to correlate with drinking iron level (Supplementary Figs. 5–8), although data was sparse at the upper end of the range. Similarly, iron and anaemia status were not associated with drinking water iron, when restricting the regression analyses to those who used tubewells/boreholes (Supplementary Table 4). Finally, there was no strong evidence of effect modification by drinking water source on the relationship between drinking water iron and iron/anaemia status (Supplementary Tables 5).

### Associations between iron status and anaemia

In multivariable regression analyses, iron deficiency was associated with a 5·92 g/L lower mean haemoglobin level (95% confidence interval, CI: -6·99, -4·86;), compared to iron replete (Table 4). Similarly, iron deficiency was associated with an adjusted prevalence ratio of 1·86 (95% CI: 1·61, 2·15) in anaemia. The adjusted prevalence ratio for moderate-severe anaemia in women who were iron deficient, compared to those who were iron replete, was 4·22 (95% CI: 2·89, 6·17) (Supplementary Table 6).

**Table 4:** Associations between iron status, haemoglobin levels and anaemia (*N* = 1498)

**Exposures**	**Haemoglobin level (g/L)**		**Anaemia**^**a**^	
	**Unadjusted mean difference (95% CI); *p*-value**	**Adjusted mean difference (95% CI); *p*-value**	**Unadjusted prevalence ratio (95% CI); *p*-value**	**Adjusted prevalence ratio (95% CI); *p*-value**
Ferritin (log_2_(μg/L))^b^	2·78 (2·39, 3·16); < 0·001	2·46 (2·05, 2·88); < 0·001	0·91 (0·90, 0·92); < 0·001	0·90 (0·89, 0·91); < 0·001
Iron status^c^				
Replete	Reference	Reference	Reference	Reference
Deficient	-6·49 (-7·51, -5·47); < 0·001	-5·92 (-6·99, -4·86); < 0·001	1·93 (1·68, 2·22); < 0·001	1·86 (1·61, 2·15); < 0·001

Linear regression models were used to assess the association between iron status (continuous or categorical) and haemoglobin, deriving mean differences. Logistic regression models were used to assess the association between iron status (continuous or categorical) and anaemia, with the prevalence ratios obtained using marginal effects and delta method for confidence intervals. Adjusted models included: Upazila, age, gestational weeks, gravidity, mid-upper arm circumference, education status, smokeless or chewing tobacco use, indoor smoke exposure, income quintile, iron folic acid use, and drinking water iron. R-squared values for adjusted models were haemoglobin (0.14, 0.13), anaemia (0.07, 0.07)

*CI* Confidence Interval

^a^Anaemia status is classified as no anaemia (haemoglobin ≥ 110 g/L) or anaemia (haemoglobin < 110 g/L)

^b^Ferritin is transformed to log base-2 due to a positively skewed distribution. The estimate represents the change (relative for anaemia, absolute for haemoglobin) associated with a two-fold increase in ferritin

^c^Iron status is classified as iron replete (ferritin ≥ 15 µg/L, or ferritin ≥ 30 µg/L in the presence of inflammation, C-reactive protein, CRP > 5 mg/L) or iron deficient (ferritin < 15 µg/L, or ferritin < 30 µg/L in the presence of inflammation, CRP > 5 mg/L)

## Discussion

The prevalence of anaemia and iron deficiency among pregnant women in Bangladesh, and the role of drinking water iron, is not well known. To address this, we conducted a cross-sectional study on 1500 pregnant women living in rural Bangladesh. This study found that 38% of the women were anaemic, including 10% who were moderately-to-severely anaemic, and 48% were iron deficient. High drinking water iron levels (≥ 2 mg/L) were measured in the primary drinking water source for 13%; however, drinking water iron did not appear to be a major determinant of iron status or anaemia in this group. Our results highlight the public health problem of anaemia and iron deficiency during pregnancy in this district of Bangladesh.

Iron deficiency was not previously regarded as a significant public health issue in Bangladesh, due to the high levels of iron in groundwater—the main drinking water source. However, we found nearly half of all pregnant women in this study were iron deficient (40% in the second trimester, 59% in the third trimester). Notably, 44% (86/197) of women with high drinking water iron (≥ 2 mg/L) were iron deficient. This prevalence is higher than previously reported, with earlier studies finding 8–27% of pregnant women to be iron deficient [20, 21]. However, these studies only included women in the early stages of pregnancy (≤ 20 weeks’ gestation), and more women may have become iron deficient as pregnancy advanced. The results of this study underscore the urgent need for intervention to address iron deficiency among pregnant women in the Narayanganj district.

Previous Bangladesh-based studies reported positive associations between drinking water iron and iron status [13, 14, 22]. In a high groundwater iron areas, daily iron intake from water was associated linked to higher serum ferritin levels in women of reproductive age [14]. Other studies found lower iron deficiency prevalence in high versus low groundwater water iron settings, [13, 22] although groundwater iron was not measured directly but based on the national hydrochemical map. In this study, conducted in a low groundwater iron setting, serum ferritin was higher with increased drinking water iron levels, but drinking water iron levels only accounted for small differences in iron deficiency. The highest levels of iron were observed in drinking water from tubewells or boreholes, used by 40% of participants, which was lower in this setting than nationally [12]. Nevertheless, no strong relationship between drinking water iron and iron status was observed among women who primarily used tubewells/boreholes. Notably, the highest drinking water iron measurement observed in this study was 7 mg/L, much lower than the 47 mg/L and 61 mg/L reported in Northern Bangladesh [23] and in the National Hydrochemical survey [11]. This suggests that drinking water iron levels do influence host iron status, but the threshold for this association is higher than concentrations observed here. Future research in settings with higher drinking water iron are required to establish this. Importantly, our findings suggest that the occurrence of iron deficiency and its contribution to anaemia may not be as consistent across Bangladesh as previously thought. Moreover, as there are large areas of low drinking water iron throughout Bangladesh, iron deficiency could be an underrecognized public health problem.

The standard of care in Bangladesh for oral iron supplementation during pregnancy involves providing a daily dose of 60 mg elemental iron for at least 180 days, preferably in combination with folic acid and other micronutrients. This protocol is recommended by the World Health Organization (WHO) and implemented by the Bangladesh Ministry of Health and Family Welfare as part of their national antenatal care program [24, 25]. However, challenges exist within Bangladesh regarding iron supplement coverage; [26] according to the 2007 Bangladesh Demographic Health Survey, 45% of women who gave birth within the last 5 years did not receive iron supplements during their pregnancy [27]. In this cohort, self-reported oral IFA supplement use was high at 71%. Despite this, 48% (95% CI: 45%, 50%) of all women were iron deficient and 38% (95% CI 35%, 40%) were anaemic. Oral iron supplements are known to have adverse gastrointestinal side effects that could influence treatment compliance, [24] so it’s possible that women in this study did not take the dose required to prevent iron deficiency and/or anaemia. Women may have also presented to their first antenatal care appointment too late in pregnancy for the oral iron to be effective, in our study 35% had not yet received antenatal care at study enrolment.

These findings highlight the importance of considering broader public health strategies. The WHO recommends iron supplementation for all menstruating women in regions where anaemia prevalence is 20% or higher, not just during pregnancy [28]. In Vietnam, this approach reduced anaemia by 19% after 12 months of iron supplementation and deworming [29]. Similarly, in certain regions of India, school-based IFA supplementation program for all adolescent girls significantly lowered anaemia within a year [30]. Given that a quarter of the pregnant women in this study were adolescents, this strategy could be highly effective here. As an alternative to oral iron, modern intravenous iron products are widely used in high income countries [31]. Multiple systematic reviews have shown that intravenous iron leads to a more rapid improvement in haemoglobin and iron stores when compared to oral iron [32–34]. In LMICs, modern intravenous iron formulations present a novel strategy to rapidly treat moderate to severe anaemia during pregnancy. Trials are currently underway in Malawi, Nigeria and India to assess its safety and efficacy [35–38]. In Bangladesh, modern intrevenous iron formulations like ferric carboxymaltose have been approved for use, however, region-specific evidence on its efficacy and feasibility is still required before adopting intravenous iron into routine antenatal care.

To our knowledge this is the largest study of anaemia in pregnant Bangladeshi women. Participants were identified through both government health facility registers and doorknocking, so we are confident that this cohort is representative of pregnant women in the Narayanganj district – a further strength of this study. However, we only captured women who were 13–32 weeks pregnant, missing those in the first trimester and late in the third trimester. This cohort was broadly similar to the overall Bangladeshi population, with similar levels of secondary education (50% in this cohort; 52% in the most recent DHS), median age at first pregnancy (18 years in this cohort and in the DHS), and teenage pregnancy rates (26% of pregnant women in this cohort; 28% in the DHS) [12]. However, the study site is more urbanized than average; 100% of participants had electricity in this cohort, compared to 82% in the DHS, and fewer women relied on tubewells/boreholes as their main drinking water source (40% here versus 96% in rural settings nationally) [12]. Importantly, drinking water iron levels in this district were lower than previous studies set in Northern Bangladesh [14, 23]. Thus, the iron deficiency and anaemia prevalence observed in this study may not be generalizable to settings with high drinking water iron. Aside from iron deficiency, there are other potential causes of anaemia that we did not assess. Vitamin A and B12 deficiencies have been associated with anaemia in pregnancy elsewhere in Bangladesh but were not measured here [21, 39]. Similarly, we did not test for haemoglobinopathies linked to anaemia like thalassemia (β-thalassemia and HbE trait), detected in 5–28% in previous Bangladesh-based surveys [40, 41]. Helminth infections are another anaemia risk factor in South Asia [42], but could not be measured here as stool samples were not collected. Finally, in the context of the ongoing transition of the study area from a predominantly agrarian to semi-urban economy, it is possible that unmeasured factors, such as dietary habits and food prices, may have influenced anaemia status. While measuring these additional risk factors could enhance our understanding of the causes of anaemia in this context, public health efforts are likely to remain focused on iron deficiency due to its high prevalence and strong association with anaemia.

## Conclusions

Reducing anaemia in pregnancy is critical to improving the health of women and their babies. In this large, cross-sectional survey of pregnant women in Narayanganj district, anaemia and iron deficiency were highly prevalent. This calls attention to iron deficiency as a potentially underrecognized public health problem in pregnant Bangladeshi women, warranting further review. A better understanding of the role of groundwater iron in the aetiology and epidemiology of anaemia in Bangladesh is also required to inform setting-specific anaemia prevention strategies.

## Supplementary Information

Supplementary Material 1.

## Data Availability

De-identified individual participant data and code book underlying the results reported in this paper will be deposited in a publicly available repository (link to be provided upon publication).

## Abbreviations

CIs: Confidence Intervals
CRP: C-reactive protein
DHS: Demographic and Health Survey
ECLIA: Electrochemiluminescence immunoassay
EPDS: Edinburgh postnatal Depression Scale
FWA: Family Welfare Assistants
FRA: Field Research Assistants
IFA: Iron-folic acid
IQR: Interquartile range
LMP: Last menstrual period
MUAC: Mid-upper arm circumference
WASH: Water, sanitation and hygiene
WHO: World Health Organization
