# Supplementary material for: High anaemia and iron deficiency prevalence among pregnant women living in low groundwater iron areas of Bangladesh
Source: BMC Public Health. 2024 Nov 6;24:3059. doi: 10.1186/s12889-024-20480-2 (PMC11539743; doi:10.1186/s12889-024-20480-2)
Supplement: Supplementary file 1 — Supplementary Material 1. [file 12889_2024_20480_MOESM1_ESM.docx]

**Supplementary Material**

Table of Contents

[Supplementary Table 1: Anaemia and iron deficiency status by Upazila 2](#_Toc177718155)

[Supplementary Table 2: Prevalence of iron deficiency by severity of anaemia (N=1498) 3](#_Toc177718156)

[Supplementary Table 3: Iron concentration in drinking water by self-reported main source (N=1500) 4](#_Toc177718157)

[Supplementary Table 4: Associations between drinking water iron levels, ferritin levels, iron deficiency, haemoglobin concentration and anaemia, by main drinking water source 5](#_Toc177718158)

[Supplementary Table 5: Effect modification of the association between drinking water iron, iron status and anaemia by drinking water source 7](#_Toc177718159)

[Supplementary Table 6: Associations between iron status and moderate-severe anaemia (N=1087) 8](#_Toc177718160)

[Supplementary Figure 1: Flow diagram illustrating the screening process, including the total number of women screened, reasons for exclusion, and the final number enrolled in the study. 9](#_Toc177718161)

[Supplementary Figure 2a: Causal diagram depicting the relationships between drinking water iron and haemoglobin levels/ anaemia during pregnancy 10](#_Toc177718162)

[Supplementary Figure 2b: Causal diagram depicting the relationships between drinking water iron and ferritin levels/ iron deficiency during pregnancy 11](#_Toc177718163)

[Supplementary Figure 3: Causal diagram depicting the relationships between ferritin levels/ iron deficiency and haemoglobin levels/ anaemia during pregnancy 12](#_Toc177718164)

[Supplementary Figure 4: Prevalence of anaemia and iron deficiency across different gestational ages 13](#_Toc177718165)

[Supplementary Figure 5: Boxplot of the relationship between drinking water iron level and serum ferritin concentration among women who use tubewells/boreholes as their main drinking water source 14](#_Toc177718166)

[Supplementary Figure 6: Boxplot of the relationship between drinking water iron level and serum ferritin concentration among in women who use piped water as their main drinking water source 14](#_Toc177718167)

[Supplementary Figure 7: Boxplot of the relationship between drinking water iron level and haemoglobin concentration among women who use tubewells/boreholes as their main drinking water source 15](#_Toc177718168)

[Supplementary Figure 8: Boxplot of the relationship between drinking water iron level and haemoglobin concentration among women who use piped water as their main drinking water source 15](#_Toc177718169)

## **Supplementary Table 1: Anaemia and iron deficiency status by Upazila**

|  | **Rupganj**  **(N=500)** | **Sonargaon**  **(N=500)** | **Bandar**  **(N=500)** |
| --- | --- | --- | --- |
| **Anaemia status** |  |  |  |
| No anaemia | 301 (60·2%; 55·9%, 64·5%) | 317 (63·4%, 59·2%, 67·6%) | 315 (63·0%; 58·8%, 67·2%) |
| Anaemia | 199 (39·8%; 35·5%, 44·1%) | 183 (36·6%; 32·4%, 40·8%) | 185 (37·0%; 32·8%, 41·2%) |
| Mild anaemia | 139 (27·8%; 23·9%, 31·7%) | 141 (28·2%; 24·3%, 32·1%) | 133 (26·6%; 22·7%, 30·5%) |
| Moderate anaemia | 60 (12·0%, 9·2%, 14·8%) | 41 (8·2%; 5·8%, 10·6%) | 51 (10·2%; 7·5%, 12·9%) |
| Severe anaemia | 0 (0·0%) | 1 (0·2%; 0·0%, 0·6%) | 1 (0·2%; 0·0%, 0·6%) |
| **Iron deficiency status ^†^** |  |  |  |
| Iron replete | 266 (53·3%; 48·9%, 57·7%) | 282 (56·5%; 52·2%, 60·9%) | 236 (47·2%; 42·8%, 51·6%) |
| Iron deficient | 233 (46·7%; 42·3%, 51·1%) | 217 (43·5%, 39·1%, 47·8%) | 264 (52·8%; 48·4%, 57·2%) |
| **Iron deficiency anaemia status^†^** | 128 (25·7%; 21·8%, 29·5%) | 106 (21·2%; 17·7%, 24·8%) | 127 (25·4%; 21·6%, 29·2%) |

Data are presented as n (%; 95% confidence interval).

Anaemia status is classified as follows: no anaemia (haemoglobin ≥110g/L), mild anaemia (haemoglobin <110 and ≥ 100g/L), moderate anaemia (haemoglobin <100 and ≥70g/L), and severe anaemia (haemoglobin <70g/L).

Iron status is classified as iron replete (ferritin≥15µg/L, or ferritin≥30µg/L in the presence of inflammation, C-reactive protein, CRP>5mg/L) or iron deficient (ferritin <15µg/L, or ferritin<30µg/L in the presence of inflammation, CRP>5mg/L).

Iron deficiency anaemia is defined as haemoglobin <110g/L combined with ferritin<15µg/L, or ferritin<30µg/L in the presence of inflammation (CRP>5mg/L).

^†^ N=1498 total, with N=499 in Rupganj, N=499 in Sonargaon, and N=500 in Bandar.

## **Supplementary Table 2: Prevalence of iron deficiency by severity of anaemia (N=1498)**

|  | **Total**  **(N=1498)** | | **Trimester 2**  **(N=897)** | | **Trimester 3**  **(N=601)** | |
| --- | --- | --- | --- | --- | --- | --- |
|  | **Iron replete** | **Iron deficient** | **Iron replete** | **Iron deficient** | **Iron replete** | **Iron deficient** |
| No anaemia | 579 (62·1%) | 353 (37·9%) | 405 (69·4%) | 179 (30·6%) | 174 (50·0%) | 174 (50·0%) |
| Anaemia | 205 (36·2%) | 361 (63·8%) | 132 (42·2%) | 181 (57·8%) | 73 (28·9%) | 180 (71·1%) |
| Mild anaemia | 173 (42·0%) | 239 (58·0%) | 111 (46·6%) | 127 (53·4%) | 62 (35·6%) | 112 (64·4%) |
| Moderate anaemia | 32 (21·1%) | 120 (79·0%) | 21 (28·8%) | 52 (71·2%) | 11 (13·9%) | 68 (86·1%) |
| Severe anaemia | 0 (0·0%) | 2 (100·0%) | 0 (0·0%) | 2 (100·0%) | 0 (0·0%) | 0 (0·0%) |

Data are presented as n (%).

Anaemia status is classified as follows: no anaemia (haemoglobin ≥110g/L), mild anaemia (haemoglobin <110 and ≥ 100g/L), moderate anaemia (haemoglobin <100 and ≥70g/L) severe anaemia (haemoglobin <70g/L).

Iron status is classified as iron replete (ferritin≥15µg/L, or ferritin≥30µg/L in the presence of inflammation, C-reactive protein, CRP>5mg/L) or iron deficient (ferritin <15µg/L, or ferritin<30µg/L in the presence of inflammation, CRP>5mg/L).

## **Supplementary Table 3: Iron concentration in drinking water by self-reported main source (N=1500)**

|  | **Drinking water iron (mg/L)** | | |
| --- | --- | --- | --- |
|  | **Median (IQR), range^‡^** | **Low (<2mg/L)** | **High (≥ 2mg/L)** |
| **All sites (N=1500)** | **0·0 (0·0-0·0), 0·0-7·0** | **1303 (86**·**9%)** | **197 (13**·**1%)** |
| Piped water source (N=895, 59·7%) | 0·0 (0·0-0·0), 0·0-7·0 | 842 (94·1%) | 53 (5·9%) |
| Piped into dwelling (N=447, 29·8%) | 0·0 (0·0-0·0), 0·0-7·0 | 420 (94·0%) | 27 (6·0%) |
| Piped into yard (N=354, 23·6%) | 0·0 (0·0-0·0), 0·0-7·0 | 335 (84·6%) | 19 (5·4%) |
| Public tap/standpipe (N=94, 6·3%) | 0·0 (0·0-0·5), 0·0-6·5 | 87 (92·6%) | 7 (7·4%) |
| Tubewell/borehole (N=605, 40·3%) | 0·0 (0·0-1·5), 0·0-7·0 | 461 (76·2%) | 144 (23·8%) |
| **Rupganj Upazila (N=500)** | **0·0 (0·0-0·0), 0·0-7·0** | **481 (96·2%)** | **19 (3·8%)** |
| Piped water source (N=439, 87·8%) | 0·0 (0·0-0·0), 0·0-6·5 | 431 (96·2%) | 8 (1·8%) |
| Piped into dwelling (N=201, 40·2%) | 0·0 (0·0-0·0), 0·0-5·0 | 199 (99·0%) | 2 (1·0%) |
| Piped into yard (N=209, 41·8%) | 0·0 (0·0-0·0), 0·0-6·5 | 203 (97·1%) | 6 (2·9%) |
| Public tap/ standpipe (N=29, 5·8%) | 0·0 (0·0-0·0), 0·0-0·0 | 29 (100·0%) | 0 (0·0%) |
| Tubewell/borehole (N=61, 12·2%) | 0·0 (0·0-1·0), 0·0-7·0 | 50 (82·0%) | 11 (18·0%) |
| **Sonargaon Upazila (N=500)** | **0·5 (0**·**0-1·5), 0**·**0-7**·**0** | **401 (80·2%)** | **99 (19·8%)** |
| Piped water source (N=154, 30·8%) | 0·0 (0·0-1·0), 0·0-7·0 | 139 (27·8%) | 15 (3·0%) |
| Piped into dwelling (N=88, 17·6%) | 0·0 (0·0-1·0), 0·0-7·0 | 76 (83·4%) | 12 (13·6%) |
| Piped into yard (N=44, 8·8%) | 0·0 (0·0-0·5), 0·0-3·0 | 43 (97·7%) | 1 (2·3%) |
| Public tap/ standpipe (N=22, 4·4%) | 0·0 (0·0-1·0), 0·0-6·5 | 20 (90·9%) | 2 (9·1%) |
| Tubewell/borehole (N=346, 69·2%) | 0·0 (0·0-1·5), 0·0-7·0 | 262 (75·7%) | 84 (24·3%) |
| **Bandar Upazila (N=500)** | **0·5 (0**·**0-1**·**0), 0**·**0-7**·**0** | **421 (84·2%)** | **79 (15·8%)** |
| Piped water source (N=302, 60·4%) | 0·0 (0·0-0·5), 0·0-7·0 | 272 (54·4%) | 30 (6·0%) |
| Piped into dwelling (N=158, 31·6%) | 0·0 (0·0-0·5), 0·0-4·0 | 145 (91·8%) | 13 (8·2%) |
| Piped into yard (N=101, 20·2%) | 0·0 (0·0-0·5), 0·0-7·0 | 89 (88·1%) | 12 (11·9%) |
| Public tap/ standpipe (N=43, 8·6%) | 0·0 (0·0-0·5), 0·0-3·0 | 38 (88·4%) | 5 (11·6%) |
| Tubewell/borehole (N=198, 39·6%) | 0·0 (0·0-1·5), 0·0-7·0 | 149 (75·3%) | 49 (24·8%) |

Data are presented as median (lower quartile – upper quartile), range or n (%).

**^‡^** Iron levels were measured in the main drinking water source using the HACH iron test kit, Model IR-18B, with an accuracy of 0.5 mg/L increments.

|  | | **Ferritin level (log_2_(μg/L)) ^a^** | | **Iron deficiency ^b^** | | **Haemoglobin (g/L)** | | **Anaemia ^c^** | | |
| --- | --- | --- | --- | --- | --- | --- | --- | --- | --- | --- |
|  |  | **Unadjusted mean difference (95% CI); p-value** | **Adjusted mean difference (95% CI); p-value** | **Unadjusted prevalence ratio (95% CI); p-value** | **Adjusted prevalence ratio (95% CI); p-value** | **Unadjusted mean difference (95% CI); p-value** | **Adjusted mean difference (95% CI); p-value** | **Unadjusted prevalence ratio (95% CI); p-value** | **Adjusted prevalence ratio (95% CI); p-value** |  |
| **Piped (N=893) ^d^** | |  |  |  |  |  |  |  |  |  |
| Drinking water iron (log_2_(mg/L)) ^e^ | | 0·10 (0·04, 0·16); 0·001 | 0·12 (0·06, 0·18); <0·001 | 0·96 (0·91, 1·01); 0·15 | 0·93 (0·87, 0·99); 0·03 | 0·30 (-0·19, 0·80); 0·23 | 0·19 (-0·33, 0·71); 0·48 | 0·98 (0·93, 1·04); 0·57 | 0·99 (0·93, 1·05); 0·68 |  |
| Drinking water iron | |  |  |  |  |  |  |  |  |  |
| Low (<2mg/L) | | Reference | Reference | Reference | Reference | Reference | Reference | Reference | Reference |  |
| High (≥2mg/L) | | 0·24 (-0·12, 0·60); 0.19 | 0·35 (0·01, 0·69); 0·04 | 0·97 (0·73, 1·29); 0·84 | 0·87 (0·64, 1·17); 0.36 | -0·92 (-3·90, 2·06); 0·54 | -0.75 (-3.65, 2.14); 0.61 | 1·08 (0·79, 1·49); 0·63 | 1·06 (0·76, 1·46); 0.74 |  |
| **Tubewell/borehole (N=605) ^d^** | |  |  |  |  |  |  |  |  |  |
| Drinking water iron (log_2_(mg/L)) ^e^ | | 0·02 (-0·03, 0·08); 0·41 | 0·04 (-0·02, 0·09); 0·20 | 0·96 (0·92, 1·02); 0·18 | 0·96 (0·91, 1·01); 0·10 | -0·01 (-0·46, 0·43); 0·95 | 0·07 (-0·39, 0·53); 0·76 | 1·00 (0·94, 1·06); 0·96 | 0·99 (0·93, 1·05); 0·76 |  |
| Drinking water iron |  |  |  |  |  |  |  |  |  |  |
| Low (<2mg/L) | | Reference | Reference | Reference | Reference | Reference | Reference | Reference | Reference |  |
| High (≥2mg/L) | | 0·08 (-0·16, 0·32); 0·50 | 0·18 (-0·05, 0·41); 0·12 | 0·94 (0·76, 1·17); 0·59 | 0·89 (0·72, 1·10); 0.27 | 0·49 (-1·44, 2·42); 0·62 | 1·05 (-0·88, 2·99); 0·29 | 1·03 (0·79, 1·33); 0·84 | 0·97 (0·75, 1·26); 0·83 |  |

## **Supplementary Table 4: Associations between drinking water iron levels, ferritin levels, iron deficiency, haemoglobin concentration and anaemia, by main drinking water source**

CI: Confidence Interval.

Unadjusted and adjusted mean differences were derived from linear regression models. Unadjusted and adjusted prevalence ratios were derived from logistic regression models, with the prevalence ratios obtained using marginal effects and corresponding confidence intervals using the delta method. Models included drinking water iron as either a discrete or categorical variable. All adjusted models included the following covariates: Upazila, age, gestational age (weeks), gravidity, mid-upper arm circumference, education status, smokeless or chewing tobacco use, indoor smoke exposure, income quintile, and iron folic acid use.

^a^ Ferritin was log base-2 transformed before fitting the model due to a positively skewed distribution.

^b^ Iron status is defined as iron replete (ferritin≥15µg/L, or ferritin≥30µg/L in the presence of inflammation, C-reactive protein, CRP>5mg/L) or iron deficient (ferritin<15µg/L, or ferritin<30µg/L in the presence of inflammation, CRP>5mg/L).

^c^ Anaemia status is classified as no anaemia (haemoglobin ≥110g/L) or anaemia (haemoglobin <110g/L).

^d^ Main drinking water source was self-reported. Piped includes piped into the house, yard or public taps.

^e^ Drinking water iron is transformed to log base-2 due to a positively skewed distribution, with observations of no detected water iron set to half the detection limit (0.125mg/L).The estimate represents the outcome change (relative for ferritin, iron deficiency, anaemia, and absolute for haemoglobin) associated with a two-fold increase in drinking water iron.

## **Supplementary Table 5: Effect modification of the association between drinking water iron, iron status and anaemia by drinking water source**

|  |  | **Ferritin ^b^ (μg/L)** | | **Iron deficiency ^d^** | | **Haemoglobin (g/L)** | | **Anaemia ^e^** | |
| --- | --- | --- | --- | --- | --- | --- | --- | --- | --- |
|  | **Drinking water source ^a^** | **Adjusted mean difference (95% CI)** | **p-value** ^c^ | **Adjusted prevalence ratio (95% CI)** | **p-value** ^b^ | **Adjusted mean difference (95% CI)** | **p-value** ^b^ | **Adjusted prevalence ratio (95% CI)** | **p-value** ^b^ |
| Drinking water iron high (≥2mg/L) vs. low (<2mg/L) | **Piped (N=895)** | 0·35 (0·01, 0·69) | 0·40 | 0·87 (0·64, 1·17) | 0.86 | -0·75 (-3·65, 2·14) | 0·30 | 1·06 (0·76, 1·46) | 0·70 |
|  | **Tubewell/ borehole (N=605)** | 0·18 (-0·05, 0·41) |  | 0·89 (0·72, 1·10) |  | 1·05 (-0·88, 2·99) |  | 0·97 (0·75, 1·26) |  |

CI: Confidence Interval.

Adjusted mean differences were derived from a linear regression model. Adjusted prevalence ratios were derived from a logistic regression model, with the prevalence ratios obtained using marginal effects and corresponding confidence intervals using the delta method. Adjusted models included the following covariates: Upazila, age, gestational age (weeks), gravidity, mid-upper arm circumference, education status, smokeless or chewing tobacco use, indoor smoke exposure, income quintile, and iron folic acid use. To evaluate effect modification by drinking water source, drinking water source and the interaction between drinking water iron and source were added to the models.

^a^ Main drinking water source was self-reported. Piped includes piped into the house, yard or public taps.

^b^ Ferritin was log base-2 transformed before fitting the model due to a positively skewed distribution.

^c^ P-value obtained from an interaction test between drinking water iron and drinking water source.

^d^ Iron status defined as iron replete (ferritin≥15µg/L, or ferritin≥30µg/L in the presence of inflammation, C-reactive protein, CRP>5mg/L) or iron deficient (ferritin <15µg/L, or ferritin<30µg/L in the presence of inflammation, CRP>5mg/L).

^e^ Anaemia status defined as no anaemia (haemoglobin ≥110g/L) or anaemia (haemoglobin <110g/L).

| **Supplementary Table 6: Associations between iron status and moderate-severe anaemia (N=1087)** | | | |
| --- | --- | --- | --- |
|  | | **Moderate-severe anaemia ^a^** | |
|  |  | **Unadjusted prevalence ratio (95% CI); p-value** | **Adjusted prevalence ratio (95% CI); p-value** |
| Ferritin (log_2_(μg/L)) ^b^ |  | 0·88 (0·84, 0·92); <0·001 | 0·83 (0·79, 0·89); <0·001 |
| Iron status ^c^ | Replete | Reference | Reference |
|  | Deficient | 4·90 (3·39, 7·10); <0·001 | 4·22 (2·89, 6·17); <0·001 |

CI: Confidence Interval.

Unadjusted and adjusted prevalence ratios were derived from logistic regression models, with the prevalence ratios obtained using marginal effects and corresponding confidence intervals using the delta method. Ferritin and iron status exposures were run in separate models. All adjusted models included the following covariates: Upazila, age, gestational age (weeks), gravidity, mid-upper arm circumference, education status, smokeless or chewing tobacco use, indoor smoke exposure, income quintile, iron folic acid use, and drinking water iron.

^a^ Anaemia status defined as no anaemia (haemoglobin ≥110g/L), or moderate-severe anaemia (haemoglobin <100g/L). Participants with mild anaemia were not included in this analysis.

^b^ Ferritin was transformed to log base-2 due to positively skewed distribution, thus the estimate represents the change associated with a two-fold increase in ferritin.

^c^ Iron status is defined as iron replete (ferritin≥15µg/L, or ferritin≥30µg/L in the presence of inflammation, C-reactive protein, CRP>5mg/L) or iron deficient (ferritin<15µg/L, or ferritin<30µg/L in the presence of inflammation, CRP>5mg/L).


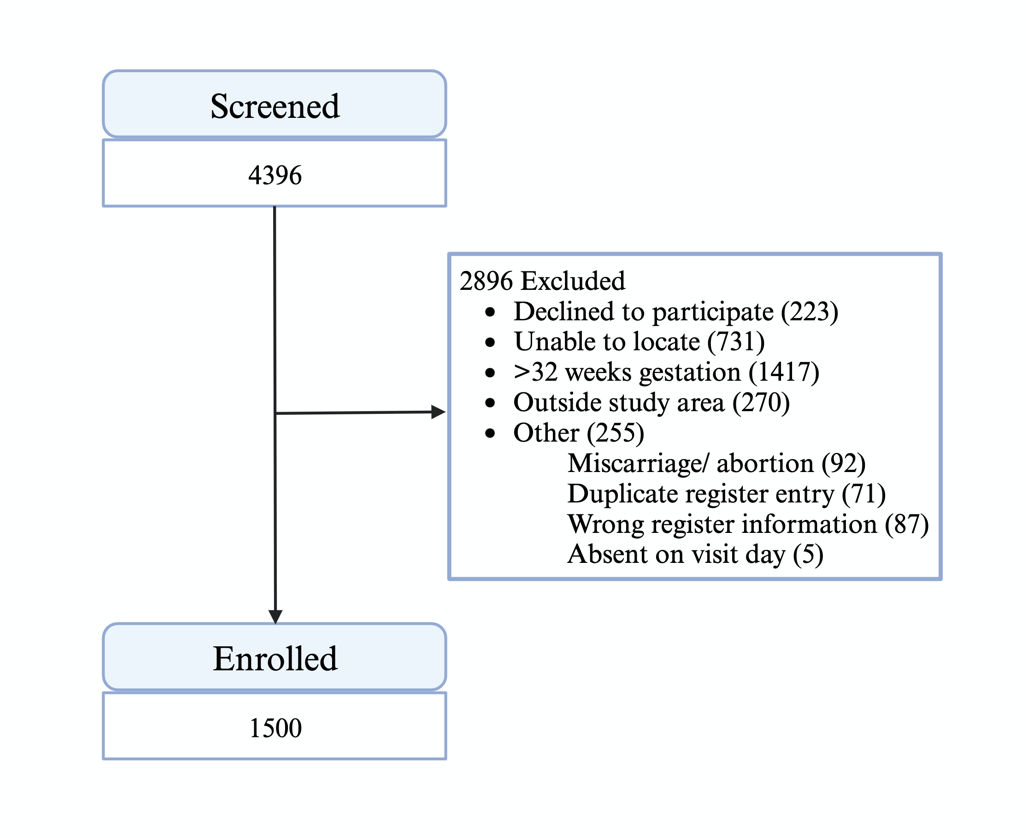


## **Supplementary Figure 1: Flow diagram illustrating the screening process, including the total number of women screened, reasons for exclusion, and the final number enrolled in the study.**


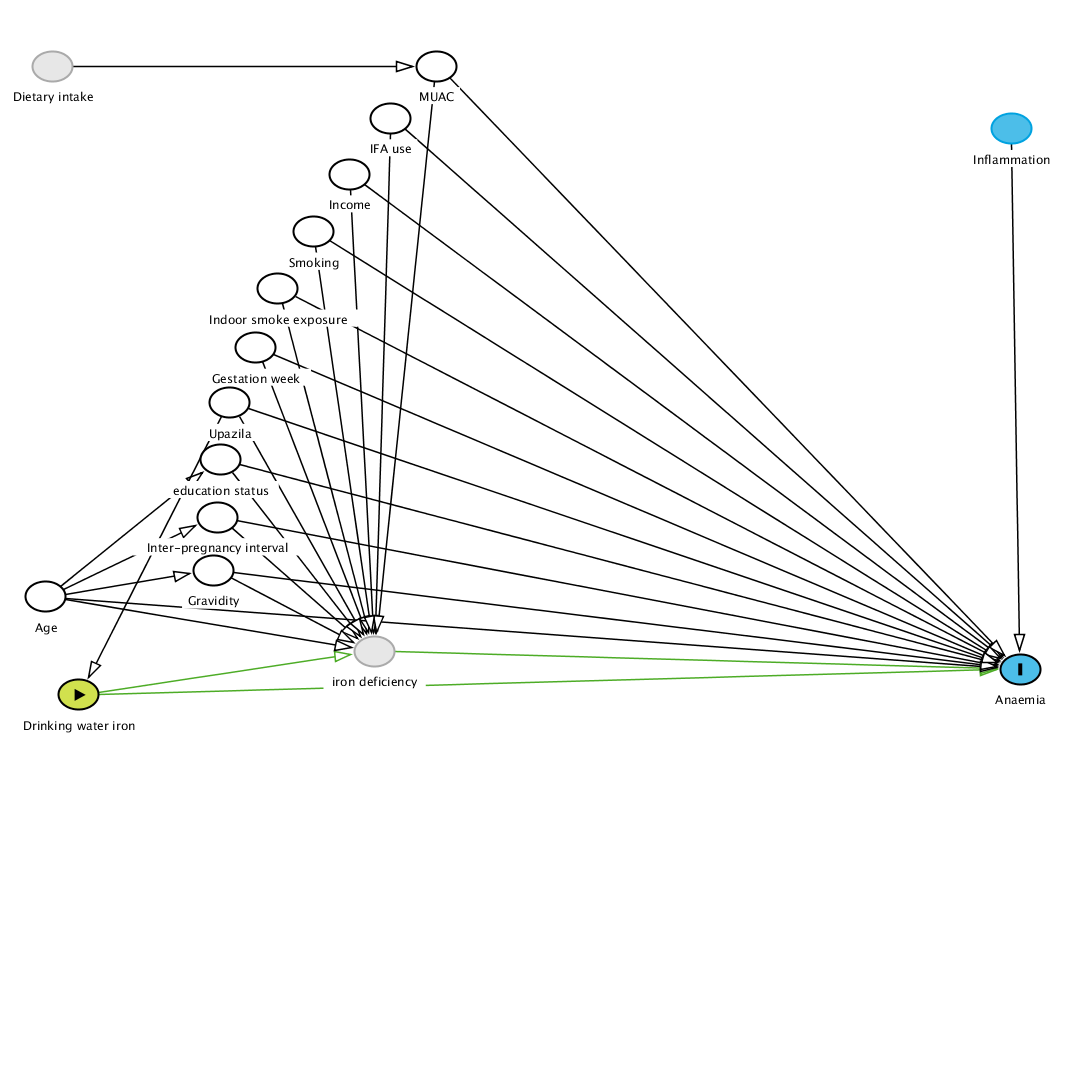


**Supplementary Figure 2a: Causal diagram depicting the relationships between drinking water iron and haemoglobin levels/ anaemia during pregnancy.** The diagram identifies the adjustment set needed to estimate the total effect of drinking water iron on haemoglobin levels/anaemia, which includes age, gravidity, inter-pregnancy interval, education status, Upazila, gestation week, smoking status, indoor smoke exposure, income, iron folic acid (IFA) use, mid-upper arm circumference (MUAC). The green arrowed node represents the exposure of interest (drinking water iron), the blue nodes denote the outcome of interest (haemoglobin levels/ anaemia) and its ancestors (inflammation), white nodes signify adjusted exposure variables, grey nodes indicate unadjusted exposure variables, and lines depict the causal paths between these variables. This diagram was created using DAGitty v3.0.


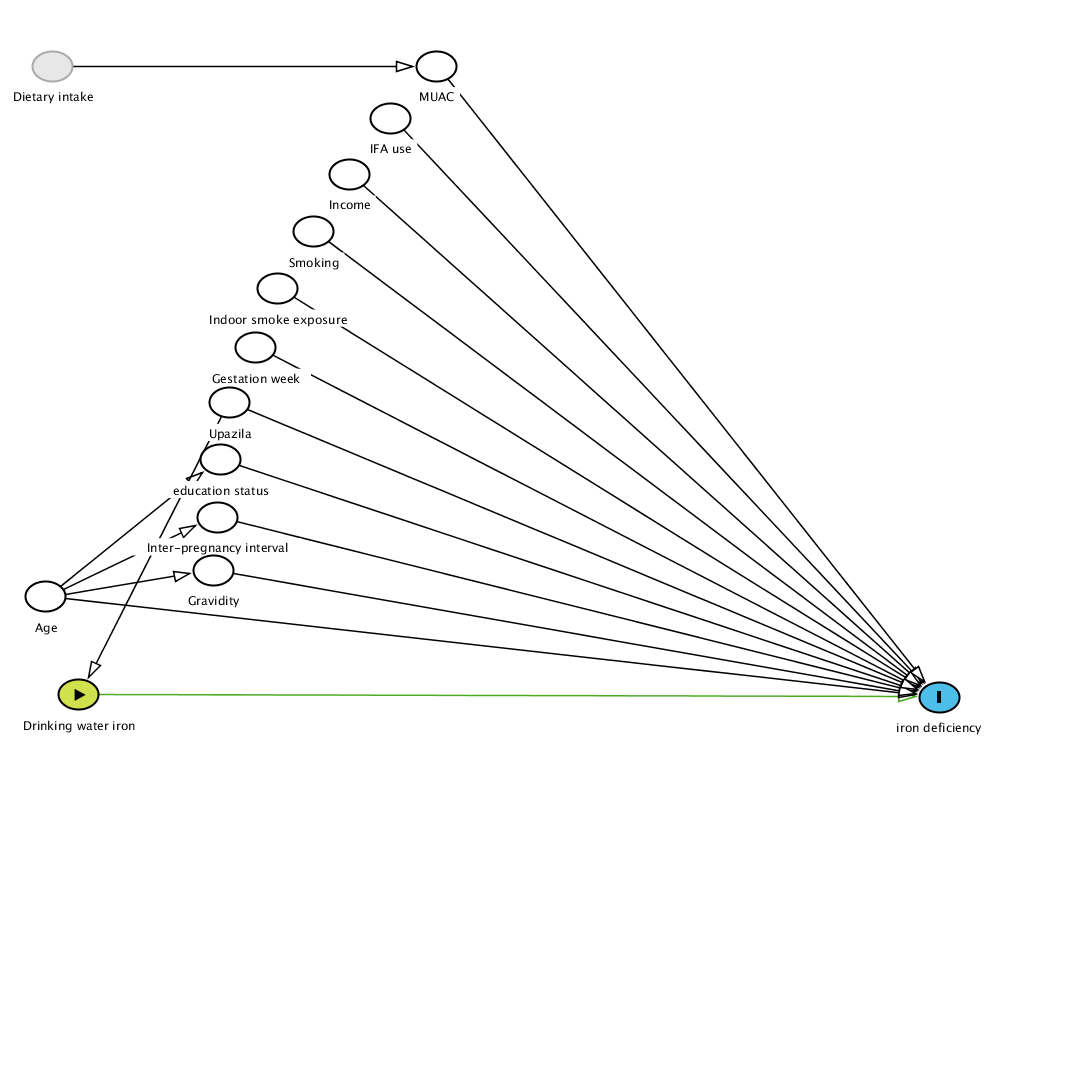


**Supplementary Figure 2b: Causal diagram depicting the relationships between drinking water iron and ferritin levels/ iron deficiency during pregnancy.** The diagram identifies the adjustment set needed to estimate the total effect of drinking water iron on ferritin levels/iron deficiency, which includes age, gravidity, inter-pregnancy interval, education status, Upazila, gestation week, smoking status, indoor smoke exposure, income, iron folic acid (IFA) use, mid-upper arm circumference (MUAC). The green arrowed node represents the key exposure of interest (drinking water iron), the blue nodes represent the outcome of interest (ferritin levels/ iron deficiency), white nodes signify adjusted exposure variables, grey nodes indicate unadjusted exposure variables, and lines depict the causal paths between these variables. This diagram was created using DAGitty v3.0.


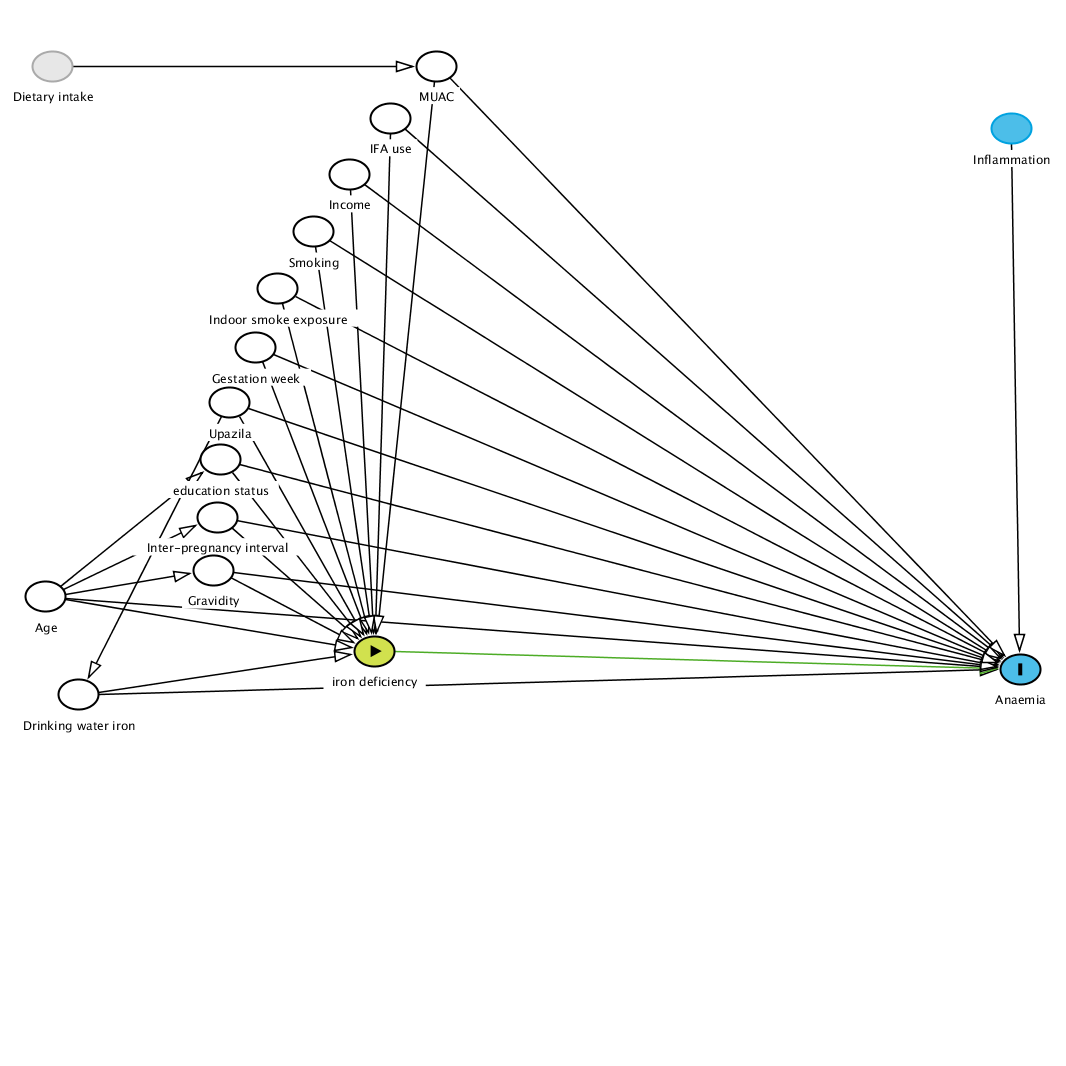


**Supplementary Figure 3: Causal diagram depicting the relationships between ferritin levels/ iron deficiency and haemoglobin levels/ anaemia during pregnancy.** The diagram identifies the adjustment set needed to estimate the total effect of drinking water iron on haemoglobin levels/anaemia, which includes drinking water iron, age, gravidity, inter-pregnancy interval, education status, Upazila, gestation week, smoking status, indoor smoke exposure, income, iron folic acid (IFA) use, mid-upper arm circumference (MUAC). The green arrowed node represents the key exposure of interest (ferritin levels/ iron deficiency), the blue nodes represent the outcome of interest (haemolgobin levels/anaemia) and its ancestors (inflammation), white nodes signify adjusted exposure variables, grey nodes indicate unadjusted exposure variables, and lines depict the causal paths between these variables. This diagram was created using DAGitty v3.0.

**Supplementary Figure 4: Prevalence of anaemia and iron deficiency across different gestational ages.**

Anaemia is defined as haemoglobin levels <110g/L. Iron deficiency is defined as ferritin levels <15µg/L, or ferritin<30µg/L in the presence of inflammation (CRP >5mg/L). Gestational age (weeks) is calculated from the first date of last menstrual period. The bars represent the estimated prevalence of anaemia (blue) and iron deficiency (orange) for each gestational age, with capped lines indicating 95% confidence intervals.

**5a 5b**

**Supplementary Figure 5: Boxplot of the relationship between drinking water iron level and serum ferritin concentration among women who use tubewells/boreholes as their main drinking water source** **(N=605).** Panel 5a displays ferritin concentrations across all measured drinking water iron levels, while panel 5b compares ferritin concentrations between low and high drinking water iron levels. Ferritin concentrations are transformed to log base-2 due to their positively skewed distribution. The boxes represent the interquartile range (25^th^ to 75^th^ percentiles), the horizontal white line within each box indicates the median, and individual dots represent the actual data points.

**6a 6b**

**Supplementary Figure 6: Boxplot of the relationship between drinking water iron level and serum ferritin concentration among in women who use piped water as their main drinking water source** **(N=895).** Panel 6a displays ferritin concentrations across all measured drinking water iron levels, while panel 6a compares ferritin concentration between low and high drinking water iron levels. Main drinking water source includes piped into the house, piped into the yard and public taps. Ferritin concentrations are transformed to log base-2 due to their positively skewed distribution. The boxes represent the interquartile range (25^th^ to 75^th^ percentiles), the horizontal white line within each box indicates the median, and individual dots represent the actual data points.

**7a 7b**

**Supplementary Figure 7: Boxplot of the relationship between drinking water iron level and haemoglobin concentration among women who use tubewells/boreholes as their main drinking water source (N=605).** Panel 7a displays haemoglobin concentration across all measured drinking water iron levels, while panel 7b compares haemoglobin concentrations between low and high drinking water iron levels. The boxes represent the interquartile range (25^th^ to 75^th^ percentiles), the horizontal white line within each box represents the median, and individual dots represent the actual data points.

**8a 8b**

**Supplementary Figure 8: Boxplot of the relationship between drinking water iron level and haemoglobin concentration among women who use piped water as their main drinking water source (N=895).** Panel 8a displays haemoglobin concentrations across all measured drinking water iron levels, while panel 8b compares haemoglobin concentration between low and high drinking water iron levels. Main drinking water source includes piped into the house, piped into the yard and public taps. The boxes represent the interquartile range (25^th^ to 75^th^ percentiles), the horizontal white line within each box represents the median, and individual dots are the actual data points.
